# Identification and Characterization of a Broadly Cross-Reactive HIV-1 Human Monoclonal Antibody That Binds to Both gp120 and gp41

**DOI:** 10.1371/journal.pone.0044241

**Published:** 2012-09-10

**Authors:** Mei-Yun Zhang, Tingting Yuan, Jingjing Li, Andrew Rosa Borges, Jennifer D. Watkins, Javier Guenaga, Zheng Yang, Yanping Wang, Richard Wilson, Yuxing Li, Victoria R. Polonis, Seth H. Pincus, Ruth M. Ruprecht, Dimiter S. Dimitrov

**Affiliations:** 1 AIDS Institute; Department of Microbiology, Li Ka Shing Faculty of Medicine, The University of Hong Kong, Hong Kong, China; 2 United States Military HIV Research Program, Department of Vaccine Research, Henry M. Jackson Foundation, Rockville, Maryland, United States of America; 3 Dana-Farber Cancer Institute, Boston, Massachusetts, United States of America; 4 Harvard Medical School, Boston, Massachusetts, United States of America; 5 International AIDS Vaccine Initiative Neutralizing Antibody Center at TSRI, Department of Immunology and Microbial Science, The Scripps Research Institute, La Jolla, California, United States of America; 6 CCRNP, Center for Cancer Research, National Cancer Institute-Frederick, National Institutes of Health, Frederick, Maryland, United States of America; 7 United States Military HIV Research Program, Division of Retrovirology, Walter Reed Army Institute of Research, Rockville, Maryland, United States of America; 8 Children's Hospital, New Orleans, Louisiana, United States of America; 9 Louisiana State University Health Sciences Center, New Orleans, Louisiana, United States of America; Shanghai Medical College, Fudan University, China

## Abstract

Identification of broadly cross-reactive HIV-1-neutralizing antibodies (bnAbs) may assist vaccine immunogen design. Here we report a novel human monoclonal antibody (mAb), designated m43, which co-targets the gp120 and gp41 subunits of the HIV-1 envelope glycoprotein (Env). M43 bound to recombinant gp140 s from various primary isolates, to membrane-associated Envs on transfected cells and HIV-1 infected cells, as well as to recombinant gp120 s and gp41 fusion intermediate structures containing N-trimer structure, but did not bind to denatured recombinant gp140 s and the CD4 binding site (CD4bs) mutant, gp120 D368R, suggesting that the m43 epitope is conformational and overlaps the CD4bs on gp120 and the N-trimer structure on gp41. M43 neutralized 34% of the HIV-1 primary isolates from different clades and all the SHIVs tested in assays based on infection of peripheral blood mononuclear cells (PBMCs) by replication-competent virus, but was less potent in cell line-based pseudovirus assays. In contrast to CD4, m43 did not induce Env conformational changes upon binding leading to exposure of the coreceptor binding site, enhanced binding of mAbs 2F5 and 4E10 specific for the membrane proximal external region (MPER) of gp41 Envs, or increased gp120 shedding. The overall modest neutralization activity of m43 is likely due to the limited binding of m43 to functional Envs which could be increased by antibody engineering if needed. M43 may represent a new class of bnAbs targeting conformational epitopes overlapping structures on both gp120 and gp41. Its novel epitope and possibly new mechanism(s) of neutralization could helpdesign improved vaccine immunogens and candidate therapeutics.

## Introduction

Development of an effective HIV-1 vaccine will likely require elicitation of broad and potent neutralizing antibody (nAb) responses against the envelope glycoprotein (Env). HIV-1 uses various mechanisms to escape human immune surveillance. Early HIV-1 infection frequently results in isolate-specific nAbs. A small percentage of HIV-1-infected individuals gradually develop broadly cross-reactive HIV-neutralizing antibodies (bnAbs) over a period of years. Profiling the specificity of bnAbs in the plasma of such “elite controllers” revealed the presence of high titers of CD4 binding site (CD4bs) Abs or bnAbs specific for other neutralizing determinants [1], [2], [3], [4]. A great effort has been made to isolate monoclonal bnAbs (bnmAbs) from such individuals. Four well studied bnmAbs, the CD4bs mAb b12 [5], the glycan-specific mAb 2G12 [6], [7], and MPER-specific mAbs 2F5 and 4E10 [8], [9], were identified more than a decade ago. Numerous new bnmAbs that are more potent than these four bnmAbs have been identified in the past two to three years, including PG9/PG16 [10], HJ16 [11], VRC01-03 [12], and very recently reported PGTs [13], VRC01-like Abs (VRC-CHs, VRC-PG04) [14], and 8ANCs, 3BNCs and 12A21 [15], etc. Most of the newly identified potent bnmAbs recognize the CD4bs or their epitopes overlap with the CD4bs. Others recognize variable loops and glycans of gp120 subunit. Co-crystallization of some of the bnmAbs with gp120 core protein or Env-derived peptides has revealed several conserved neutralizing epitopes [6], [15], [16], [17], [18], [19], [20], [21]. Both b12 and VRC01 used their heavy chain complementarity-determining region 2 (HCDR2) to mimic CD4 binding to gp120 with some differences. A critical b12 residue mimics the interaction of the CD4 phenylalanine (Phe43_CD4_), while a critical VRC01 residue mimics the interaction of the CD4 arginine (Arg59_CD4_) with gp120 [16], [17]. Two genetically related bnmAbs, PG 9/16, have one of the longest HCDR3 (28 amino acids, AA) observed for human antibodies and preferentially recognize the oligomeric conformation of the Env. PG9 binds to a site of vulnerability comprising of two conserved glycans and one of the four β-strands formed by the V1/V2 loops with the long protruding H3 loop penetrating the glycan shield [19]. Similarly, PGT 127 and 128 bind to a high mannose site on gp120, and the antibody penetrates the glycan shield and recognizes two conserved glycans and a short β-strand segment of the V3 loop [21]. PG16 and the recently reported CH01-CH04 and PGT145 may share this binding mode of glycan penetration by extended anionic loops. 2F5 and 4E10 recognize linear epitopes on the MPER of gp41 that is thought to play a key role in the fusion process [8], [9]. 2G12 is a unique bnmAb that has a domain-swapping feature and recognizes a cluster of oligomannose residues on gp120 [6], [7]. These neutralizing epitopes may be used for vaccine immunogen design although such effort based on the epitopes of b12, 2F5, 4E10 and 2G12 has not been successful. Identification of novel bnmAbs may reveal new neutralizing determinants and facilitate immunogen design.

Among the above mentioned bnmAbs, b12 was the only mAb isolated by antibody phage display [5]. PG9/16, VRC01-03 and PG9/16-like Abs were isolated by single memory B-cell-sorting in combination with high-throughput screening for bnAb-secreting B cells [12], [22]. 2F5, 4E10 and 2G12 were isolated by conventional Epstein-Barr Virus (EBV)-mediated immortalization of antibody-secreting B cells, while HJ16 was isolated by using an improved EBV-mediated memory B cell immortalization method in combination with high-throughput parallel screening with a panel of recombinant Env-based antigens. We developed the competitive antigen panning (CAP) methodology for isolation of gp41-specific mAbs that bind to Env. After panning a phage-displayed immune antibody Fab library by CAP and screening the panned libraries for gp41-specific mAbs, we found one mAb, designated m43, which bound to both gp120 and gp41, as well as to recombinant gp140s. We extensively characterized m43 for binding and neutralizing activities and possible mechanism of neutralization. M43 exhibited unique features in binding to Env trimers and may represent a new class of bnAbs.

## Materials and Methods

### Cells, viruses, plasmids, gp120, gp140, gp41Fc fusion protein, peptides and antibodies

This study was approved by the ethics committee of Walter Reed Army Institute of Research (WRAIR) IRB (FWA#00000015/IRB00000794). Initial WRAIR IRB approval was on 5 March 2008. Written consent was received by all participants of this study. 293T cells were purchased from ATCC. Free-style 293 cells were purchased from Invitrogen. TZM-bl cell line and HIV-1 isolates were obtained from the NIH AIDS Research and Reference Reagent Program (ARRRP). JC-53 and JC-10 cells were kindly provided by Dr. David Kabat [23]. H9/NL4-3 is a derivative of the CD4+ human lymphoma cell line H9 that has been infected with the NL4-3 isolate of HIV-1. These cells maintain complete infection and, through repeated passages in tissue culture, produce infectious virus [24]. C8166.R5 cells are CD4+ lymphoma cells that have been transfected to express CCR5 [25]. Recombinant gp140s from primary isolates for ELISA along with recombinant gp140/120_89.6_, gp140/120_CM243_ and gp140/120_R2_ for panning and screening were generously provided by Dr. Christopher C. Broder (USUHS, Bethesda, Maryland). The cleavage-competent JRFL gp160 plasmid was kindly provided by Dr. Joseph Sodroski. Wild-type gp41Fc fusion protein (gp41_89.6_ fused to human Fc) was produced in our laboratory by transient transfection of free-style 293 cells using 293fectin as transfection reagent (Invitrogen). The human mAbs 2F5 and 4E10 were kindly provided by Dr. Herman Katinger. The 6-helix bundle (6HB)-specific mouse mAb NC-1 and 5-helix bundle (5HB) were kindly provided by Dr. Shibo Jiang (Fudan University, China). N36 and C34 were provided by Dr. Robert Blumenthal (NCI, NIH). Peptides DP178 and #7010 were kindly provided by Dr. Lai-Xi Wang (University of Maryland). MAbs m14, m18, m43, m44, m46, m48, b12, VRC01, and Z13 were produced in our laboratories. Fm-6 was provided by Dr. Wayne Marasco (Dana-Farber Cancer Institute). CD4-IgG2 was a kind gift of William Olson. Human mAbs 2.2b, A32, E51, and 17b were provided by Dr. James Robinson. 7B2 is a human mAb directed against the gp41/gp120 interaction region [26], as is 2.2b; CD4-IgG2 is a CD4/Ab chimera in which H and L chain V domains are replaced with CD4 domains 1 and 2 [27]; E51, 17b, and A32 are human mAbs directed against conserved epitopes of gp120. 924 is a murine mAb directed against the V3-loop of the IIIB isolate of HIV [28]. The anti-V3 mAb 39F was obtained from ARRRP. The plasmid encoding Z13 was provided by Drs. M. Zwick and D. Burton (The Scripps Research Institute). Anti-p24 mAb (183-12H-5C) and HIV immunoglobulin (HIVIG) were obtained from the NIH ARRRP. The following antibodies were purchased: Affinity Purified Sheep Anti-HIV-1-gp120 polyAb D7324 (AALTO, Dublin, Ireland), HRP-conjugated monoclonal mouse anti-M13 antibody (Pharmacia, Uppsala, Sweden), HRP-conjugated polyclonal anti-human IgG, F(ab′)_2_ antibodies (Jackson ImmunoResearch, Westgrove, PA), HRP-conjugated streptavidin (Zymed Laboratories Inc., San Francisco, CA), Phycoerythrin (PE)-conjugated polyclonal anti-human IgG, F(ab′)_2_ antibodies and PE-conjugated streptavidin (Jackson ImmunoResearch, Westgrove, PA).

### Competitive Antigen Panning (CAP) and screening

The phage library was constructed using pComb3H phagemid vector and 30 ml of bone marrow obtained from three long-term nonprogressors (A, H and K) [29] whose sera exhibited the broadest (against six primary isolates [30]) and most potent (against JR-FL at 1∶40 dilution) HIV-1 neutralization amongst the 37 HIV-1-infected individuals [31]. CAP was performed as described previously [32]. Briefly, the phage library (5×10^12^ cfu/ml) was preabsorbed on streptavidin-M280-Dynabeads in PBS for 1 h at room temperature (RT) and incubated with 50 nM biotinylated HIV-1 CM243 (clade E) Env gp140_CM243_ and 250 nM non-biotinylated gp120_CM243_ (5-fold more on molar level than biotinylated gp140_CM243_) for 2 h at RT with gentle agitation. Phage particles binding to biotinylated Env were separated from the phage library using streptavidin-M280-Dynabeads and a magnetic separator (Dynal). After washing 20 times with 1 ml of PBS containing 0.1% Tween-20 and another 20 times with 1 ml of PBS, bound phage particles were eluted from the beads using 100 mM Triethanolamine followed by neutralization with 1 M, pH7.5 Tris-HCl. For the 2^nd^ round of panning, 10 nM (2 nM for the 3^rd^ round) of biotinylated gp140_CM243_ was used as antigen and a 5-fold excess of non-biotinylated gp120_CM243_ for competition depletion. After 3^rd^ round of panning against 2 nM biotinylated gp140_CM243_ in the presence of 10 nM non-biotinylated gp120_CM243_, 96 individual clones were screened for binding to gp140_CM243_, gp120_CM243_, and gp41Fc fusion protein by phage ELISA.

### Binding assays with recombinant Env glycoproteins or peptides

ELISAs of soluble Fab m43 to recombinant HIV-1 gp140s, gp120 and gp41Fc fusion protein were performed by directly coating gp140s on 96-well plates followed by addition of three-fold serially diluted soluble Fab m43. Bound Fab m43 was detected using HRP-conjugated anti-human IgG, F(ab′)_2_ (1∶5,000) and ABTS substrate. In cases using denatured gp140s, purified gp140s were diluted in 1% sodium dodecyl sulfate-50 mM DTT to 10 µg/ml and boiled for 5 min, then diluted 1∶10 in PBS and coated on 96-well plates. Competition ELISA was performed by directly coating recombinant gp140_89.6_ followed by addition of serially diluted competitors, either gp41-derived peptides or fusion intermediate structures or HIV-1 mAbs, and a fixed concentration of non-biotinylated HIV-1 mAbs or biotinylated IgG_1_ m43 that leads to 50–70% maximum binding. Bound Fabs (m43 and m44) and IgG_1_s (2F5 and 4E10) were detected using HRP conjugated to anti-human IgG, F(ab′)_2_ (1∶5,000) and ABTS substrate. Bound mouse mAbs NC-1 was detected using HRP-conjugated anti-mouse (H+L) (1∶5,000) and ABTS substrate. Bound biotinylated IgG_1_ m43 was detected by HRP-avidin (1∶10,000) and ABTS substrate. Binding to gp120 loop deletion mutants and site mutants were carried out by coating sheep anti-HIV-1 poly Ab D7324 (at 5 µg/ml) to capture WT gp120 or mutants in culture supernatant followed by addition of serially diluted mAbs in duplicates. Western blots with gp41 intermediate structures were done as previously described [33], [34], [35], [36]. N_CCG_-gp41 (13), N35_CCG_-N13, and N34_CCG_, as well as single chain protein of five helix bundle (5HB) were recombinant polypeptides. N36/C34 -formed six helix bundle (6HB) was prepared by equally (on molar ratio) mixing synthesized linear peptide N36 and C34 followed by incubation at RT for 30 min.

### Flow cytometry

Using PEI as transfection reagent, 293T cells were cotransfected with cleavage-competent Env-expressing plasmid or cleavage-incompetent Env-expressing plasmid, JRFL or Yu2 gp160dCT/pSVIII, and HIV-1 Tat-expressing plasmid pCTAT. Six hrs posttransfection, the medium was changed to complete medium (DMEM with 10% FBS). 48 hrs posttransfection, cells were detached using trypsin-free cell dissociate buffer and washed with PBS. 2×10^5^ cells were aliquoted to each microcentrifuge tube for each ligand at a given concentration. The tubes containing the cells were subjected to centrifugation at 350× g for 5 min at RT. The cell pellets were resuspended in 100 µl Fluorescence-activated cell sorting (FACS) buffer containing the ligands and incubated at RT for 60 min. Following another two times washing, the cells were incubated with appropriate 2^nd^ antibody in100 µl FACS buffer at RT for 60 min. The stained cells were analyzed by FACS on a Beckman Coulter Calibur Instrument. Indirect immunofluorescence with persistently infected H9/NL4-3 cells or C8166.R5 cells five days after acute infection with the R5 tropic HIV isolates Ba-L or 208/K8was performed by incubating cells for 1 hr with primary Abs diluted to 10 µg/ml in PBS containing 1% BSA and 0.01% sodium azide (PBA). Where indicated, sCD4 (a gift of Upjohn) was added at 500 ng/ml. After the primary incubation, cells were washed twice then incubated with FITC or Alexa 488-conjugated anti-mouse or anti-human Ig (3 µg/ml) (Invitrogen). Following another set of washes, the cells were fixed with 2% paraformaldehyde. 10,000 cells per run were analyzed on an LSRII flow cytometer. Ab interactions were studied by incubating cells with unconjugated Abs (10 µg/ml) in PBA for 1 hr, then adding directly-labeled Ab (3 µg/ml) to the cells. After 4 hrs, the cells were washed, fixed in 2% paraformaldehyde and studied by flow cytometry.

### HIV-1/SHIV neutralization assays

Three neutralization assays based on infection of PBMCs were carried out in this study. The PBMC-p24 assay was carried out using the protocol previously described [37]. PBMCs isolated from the blood of healthy donors were plated in 75-cm^2^ plastic flasks and incubated at 37°C for 2 hrs. The nonadherent cells were collected and resuspended at 5×10^6^ in 10 ml of RPMI 1640 medium containing 10% FBS, 5 µg/ml of phytohemagglutinin (PHA), and 100 U/ml of IL-2, followed by incubation at 37°C for 3 days. The PHA-stimulated cells were infected with the corresponding primary HIV-1 isolates at a multiplicity of infection (MOI) of 0.01 in the absence or presence of an antibody at graded concentrations. Culture media were changed every 3 days. The supernatants were collected seven days post infection and tested for p24 antigen by ELISA. The percent inhibition of p24 production was calculated by using the software Calcusyn. The PBMC-RT assay was carried out as follows: 100 µl of antibodies diluted in complete RPMI with IL-2 were incubated with 50 µl of virus containing 100 TCID_50_ for 30 min at 37°C and added to 50 µl of PHA-activated PBMCs (1×10^6^) in complete RPMI 1640 with IL-2. The calculated neutralization activities refer to the antibody concentration present during this incubation step. Triplicate samples were taken on day 7 for the RT assay and percentage neutralization was calculated by dividing the RT activity of the tested samples by the RT activity of mock culture without antibody; AZT was used as a positive control with 100% inhibition at a concentration equal to 0.1 µM, where the measured cell toxicity was undetectable (IC_50_ = 0.002 µM, TCID_50_>1 µM). The SHIV neutralization assays were performed in human PBMCs followed by SIV p27 readout. The mAbs were tested against SHIV-1157ipd3N4 [38], SHIV-1157ipEL-p [39], SHIV-2873Nip [40] and SHIV_SF162P4_ [41], as previously described [38], except that polymyxin B (15 µg/ml) was added to block potentially present endotoxin [42]. The assay was repeated twice.

Three cell lines, TZM-bl, JC-10, and JC-53, were used in cell line-based pseudovirus assays. The TZM-bl cell line-based assay was carried out in triplicate by using an HIV-1 Env pseudotyping system and TZM-bl target cells containing a Tat-inducible luciferase reporter and expressing CD4, CCR5, and CXCR4. The degree of virus neutralization by antibody was achieved by measuring luciferase activity as described previously (assay #2 in [43]). Percentage of virus neutralized was calculated as the number of isolates neutralized by the mAb over the total number of isolates tested. JC-10 and JC-53 cell line-based assay is very similar to TZM-bl cell line-based assay except the target cells are changed from TZM-bl cells to JC-10 or JC-53 cells that express different level of coreceptor CCR5 [23], [44]. VSV-G pseudotype controls were included in the assays. IgG_1_ m43 did not neutralize VSV-G pseudotype virus.

### HIV-1 Env gp120 shedding

HIV-1 Env shedding assay was performed as previously described [45]. About 8 million 293T cells were seeded and transfected with 40 µg of plasmid JRFLgp160dCT/pSVIII along with 2 µg of Tat-expressing plasmid pCTAT, with Fugene6 (Roche) as transfection reagent per the manufacturer's instructions. 48 hrs post transfection, the cells were dislodged from the cell culture flask by incubating with PBS/5 mM EDTA and gently rocking at RT for 5 min. Cells were then washed with PBS and resuspended at density of to 1×10^7^ cells/ml in PBS. 200 ul of the cell suspension (about 2 million cells) were aliquoted into micro-centrifuge tubes for the shedding assay for each antibody/ligand. The cells were pelleted by centrifugation at 335× g at RT for 5 min. 160 µl of the supernatant was removed and 20 µl of the ligand stock solution were added to the cell pellets to achieve a final concentration of 50 µg/ml. The cells and ligand were mixed well and incubated at 37°C for 1 hr with gentle mixing every 20 min. The cells were then pelleted by centrifugation, and the supernatants were transferred to fresh tubes. 30 µl of each supernatant was loaded onto SDS-PAGE gels and run for 1.5 hrs at 200 V. The proteins from the supernatant were transferred from the gel to nitrocellulose membranes for Western blotting analysis. To identify shed gp120, anti-gp120 human mAb 39F (V3-specific) was used as a primary antibody (1 µg/ml) in PBS/0.1% Tween-20, and HRP-conjugated anti-human polyclonal antibodies as secondary antibody (1∶10,000), followed by development with Immobilon Western Chemiluminescent HRP substrate (Millipore).

## Results

### Isolation of a broadly cross-reactive human mAb co-targeting gp120 and gp41

**Figure 1:**
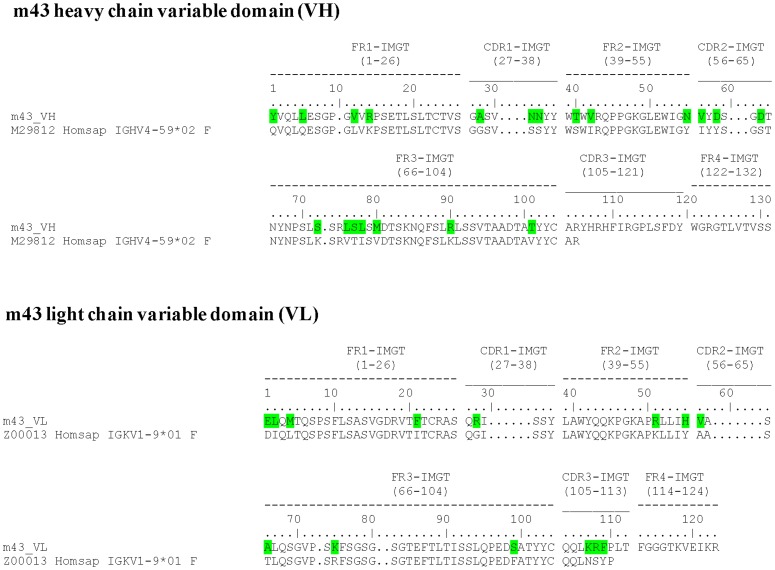
m43 sequence and extent of affinity maturation. The sequence of m43 is shown along with nearest VH- and VΚ-genomic precursors for heavy and light chain V-segments, respectively. Affinity maturation changes in V-segments are indicated in green.

Panning of a phage-displayed antibody library against recombinant gp140 often results in enrichment of gp120-specific antibodies. To identify antibodies specific for gp41 in the context of the Env, we developed the CAP methodology [32], [46] and isolated a panel of broadly cross-reactive gp41-specific mAbs, including m44 [33], m46 [47], and m48 [32]. Using biotinylated recombinant gp140_CM243_, gp140_89.6_ and gp140_R2_ in the presence of corresponding 5-fold more non-biotinylated recombinant gp120_CM243_, gp120_89.6_ and gp120_R2_, we carried out three rounds of CAP against each antigen. During screening using the gp140_CM243_-panned library for gp41-specific monoclonal phage, we found one clone, designated m43, which strongly bound all antigens used in monoclonal phage ELISA, including recombinant gp140_CM243_, gp120_CM243_, and 89.6 gp41Fc fusion protein. Fab m43 was not enriched in the panned libraries against gp140_CM243_ and was not found in 192 individual clones from the panned libraries against gp140_89.6_ and gp140_R2_, which is most likely due to the excessive amount of respective recombinant gp120s used for competition depletion. Sequence analysis showed that m43 had extensive somatic maturation in both heavy and light chain variable regions (VH and VL) (Fig. 1).

### Characterization of the m43 epitope

**Figure 2:**
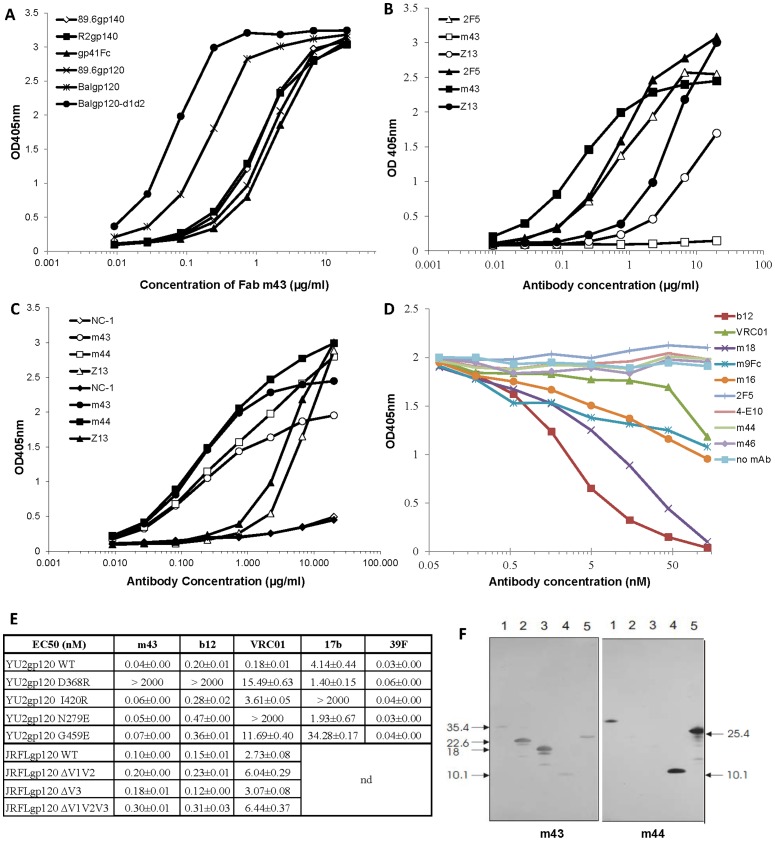
Binding of m43 and control mAbs to recombinant Envs by ELISA (A–E) and to gp41 intermediate structures by Western blot (F). **A**: Binding of Fab m43 to recombinant gp140s, gp120s and gp41Fc fusion protein. **B**: Binding of Fabs m43 and Z13, and IgG_1_ 2F5 to non-denatured (solid symbols) and denatured gp140_89.6_ (empty symbols). **C**: Binding of Fabs m43, m44 and Z13, and mouse mAb NC-1 to recombinant gp140_89.6_ in the absence (solid symbols) or presence (empty symbols) of sCD4 (2 µg/ml). **D**: Competition of CD4bs mAbs b12, m18 and VRC01, CD4i mAbs m16 and m9Fc fusion protein, and gp41-specific mAbs m44, m46, 2F5 and 4E10 with biotinylated IgG_1_ m43 for binding to coated gp140_89.6_. **E**: EC_50_s of IgG_1_ m43, CD4bs mAbs b12 and VRC01, CD4i mAb 17b, and V3 loop-specific mAb 39F with gp120_JRFL_ wild-type (WT) and V1, V2, V3 loop deletion mutants, and gp120_yu2_ WT and its site mutants in CD4bs (D368R), CD4i (I420R), loop D (N279E) and V5 loop (G459E). nd: not done. **F**: Binding of Fabs m43 and m44 to non-reduced N35_CCG_-gp41 (lane 1), N35_CCG_-N13 (lane 2), N34_CCG_ (lane 3), gp41 core (lane 4), and 5HB (25.4 kDa) (lane 5) by Western blot.

We expressed Fab m43 and characterized it for binding activity. Soluble Fab m43 bound to a panel of recombinant gp140s derived from different clades with affinities ranging from 1–30 nM except the recombinant gp140 derived from clade A isolate, UG037.8 (Table 1), suggesting that m43 epitope is conserved among gp140s. Fab m43 also bound to various recombinant gp120s, as well as to 89.6 gp41Fc fusion protein (Fig. 2A). Fab m43 bound to recombinant gp120_BaL_ fused to the first two domains of CD4 (BaLgp120-d1d2) with affinity higher than that for BaL gp120 alone (Fig. 2A). But Fab m43 did not bind to denatured recombinant gp140_89.6_ (Fig. 2B), indicating a conformational nature of the m43 epitope. Soluble CD4 (sCD4) weakly inhibited Fab m43 binding to recombinant gp140_89.6_, while it did not affect the binding of gp41-specific human mAbs m44, Z13, and mouse mAb NC-1 to recombinant gp140_89.6_ (Fig. 2C). In an attempt to localize the m43 epitope, we performed a competition ELISA of Fab m43 with N36/C34-formed six helix bundle (6HB), single-chain protein of five helix bundle (5HB), gp41-derived peptides N36, C34, and 2F5 epitope containing (underlined) peptides DP178 (YTSLIHSLIEESQNQQEKNEQELLELDKWASLWNF), and #7010 (NEQELLELDKWASLWNWFD). None of these competitors inhibited Fab m43 binding to coated recombinant gp140_89.6_ (data not shown). We then performed another competition ELISA of IgG_1_ m43 with a panel of HIV-1 mAbs for binding to coated recombinant gp140_89.6_ (Fig. 2D). IgG_1_ m43 competed strongly with CD4bs mAbs b12 and m18, and modestly with CD4bs mAb VRC01 and CD4 induced (CD4i) mAbs m16 and m9Fc fusion protein. IgG_1_ m43 did not compete with gp41-specific mAbs m44, m46, and MPER-specific mAbs 2F5 and 4E10, which is consistent with the result obtained from the competition ELISA of Fab m43 with peptides DP178 and #7010. We further measured the binding of IgG_1_ m43 to gp120_JRFL_ V1, V2, V3 loop deletion mutants, and to four gp120_yu2_ site mutants with knock-out mutations in the CD4 binding site (D368R), the coreceptor binding site (I420R), loop D (N279E), and V5 loop (G459E) in comparison with CD4bs mAbs b12 and VRC01, CD4i mAb 17b, and V3 loop-specific mAb 39F (Fig. 2E). Results showed that deletion of V1, V2 or V3 loop did not affect m43 binding to gp120. Site mutations in the coreceptor binding site, loop D or V5 also did not affect m43 binding to gp120, but site mutation in CD4bs (D368R) abolish m43 binding to gp120_YU2_. All these results indicate that m43 epitope may involve the CD4bs on gp120. The competition between IgG_1_ m43 and CD4i mAbs may be due to steric restrictions. To localize m43 epitope on gp41, we measured the binding of Fab m43 to fusion intermediate structures, including N36/C34-formed 6HB, single-chain protein of 5HB and the N-trimer-containing polypeptides N_ccg_-gp41 [34], N35_ccg_-N13 [35], and N34_ccg_ [35] by Western blot in comparison with Fab m44 [33], [36] (Fig. 2F). Fab m43 bound to N35_ccg_-N13 and N34_ccg_ containing N-trimer structure (lane 2 and 3), suggesting that the N-trimer structure of gp41 may be involved in m43 binding. Fab m43 did not bind to the 5HB (lane 5) and the 6HB (lane 4), nor to the N_ccg_-gp41 (lane 1) that comprises 6HB and N-helix. As we reported previously, Fab m44 bound to 5HB and 6HB, as well as N_ccg_-gp41 on Western blot [33]. Taken together, these results suggest that m43 binds to a conserved and conformational epitope that may involve the CD4 binding site on gp120 and the N-trimer structure on gp41.

**Table 1:** Binding of Fab m43 to recombinant gp140s from primary isolates.

Isolate	Clade	EC50, nM
		Fab m43
UG037.8	A	500 ± 291.5
tethered 89.6	B	3 ± 0.8
R2	B	3.5 ± 0.7
MW965.26	C	12.3 ± 8.3
GXC-44	C	1.5 ± 0.2
CRII92UG024.2	D	5.4 ± 0.2
GXE-14	E	1.9 ± 0.2
93BR019.10	FB	35.9 ± 14.3
92UG975.10	G	1.7 ± 0.4

Recombinant gp140s from primary isolates were directly coated on Maxisorp plates. Bound Fab m43 was detected by using HRP conjugates to anti-human IgG, F(ab′)2 as secondary antibody and ABTS as substrate. EC_50_ is the concentration at which antibody has half-maximum binding.

### Existence of the m43 epitope on functional Env trimers

**Figure 3:**
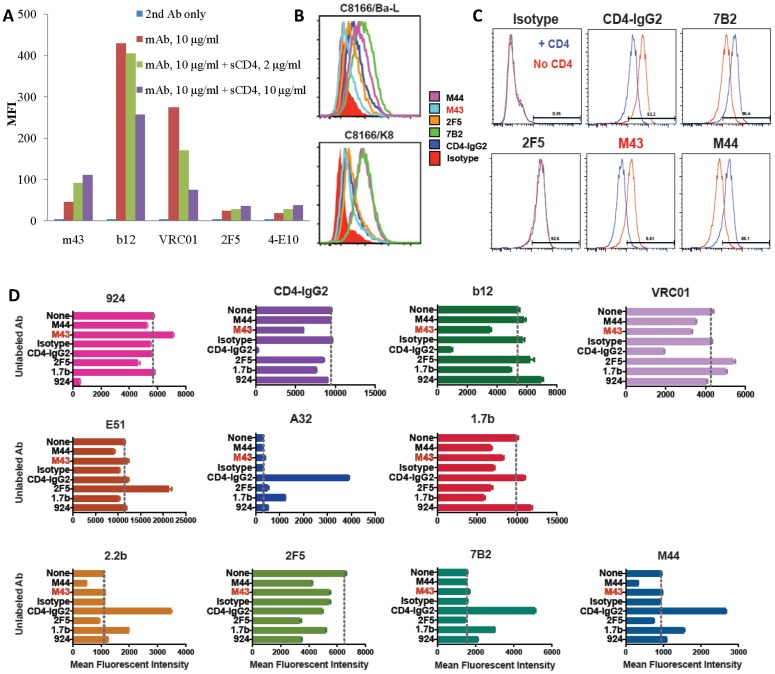
Binding of IgG_1_ m43 and other mAbs to functional Env trimers by flow cytometry. A. Binding of IgG_1_s m43, b12, VRC01, 2F5 and 4E10 to cleavage-competent gp160_JRFL_ on 293T cells. **B**. Binding of Abs to C8166.R5 cells five days after acute infection with the R5-tropic HIV isolates BaL or 208/K8. **C**. Binding of mAbs (3 µg/ml) to persistently infected H9/NL4-3 cells in the presence or absence of sCD4 (500 ng/ml). **D.** The ability of seven different unconjugated mAbs to alter the binding of eleven Alexa 488-labeled Abs to H9/NL4-3 cells. The results are displayed as the mean and SEM fluorescent intensity, with each graph representing one labeled Ab. The vertical line on each graph indicates the binding of the labeled Ab in the absence of unlabeled Ab. Fluorescent intensity values for unlabeled cells were 260±3, and for cells incubated with an Alexa 488-labeled isotype control 298±2.

We tested IgG_1_ m43 for binding to functional Env trimers by flow cytometry in comparison with IgG_1_s b12, VRC01, 2F5 and 4E10 (Fig. 3A). M43 bound to cleavage-competent JRFL gp160 on 293T cells, and its binding was slightly enhanced by sCD4. The binding of 2F5 and 4E10 to cleavage-competent JRFL gp160 was lower than that of m43, and their binding was also slightly enhanced by sCD4 (Fig. 3A). Both b12 and VRC01 showed strong binding to cleavage-competent JRFL gp160. As expected, sCD4 inhibited both b12 and VRC01 binding (Fig. 3A). We compared IgG_1_ m43 binding to cleavage-competent JRFL gp160 with its binding to cleavage-competent Yu2 gp160 and cleavage-incompetent JRFL and Yu2 gp160s by flow cytometry in comparison with 2F5 and 4E10 (Table 2). Both IgG_1_ m43 and 4E10 showed significantly higher binding to cleavage-competent Yu2 gp160 than to cleavage-competent JRFL gp160, while 2F5 binding to both cleavage-competent JRFL and Yu2 gp160s were almost the same. All three mAbs showed increased binding to cleavage-competent JRFL gp160 in the presence of sCD4. sCD4 enhanced m43 the most (about 2-fold increase) and, to less extent, 2F5 and 4E10 in binding to cleavage-competent JRFL gp160. All three mAbs showed higher binding to cleavage-incompetent JRFL and Yu2 gp160s than to the cleavage-competent counterparts, but the changes with m43 were more prominent (about 5-fold increases) than those with 2F5 and 4E10 (about 2-fold increases). sCD4 also slightly enhanced 2F5 and 4E10 binding to cleavage-competent Yu2 gp160s, while sCD4 weakly inhibited m43 binding to cleavage-competent Yu2 gp160 (Table 2). These results suggest that m43 bound better than 2F5 and 4E10 to functional Env trimers, and the binding of m43 and 2F5 was more conformation-dependent than 4E10.

**Table 2:** Binding of IgG_1_s m43, 2F5 and 4E10 to cleavage-competent and cleavage-incompetent JRFL and Yu2 gp160s on 293T cells in the absence and presence of 10 µg/ml sCD4 by flow cytometry.

mAbs	MFI			
	JRFL(+)	JRFL(−)	Yu2(+)	Yu2(−)
2nd Ab only	6.0	4.8	7.2	6.3
m43	46.8	183.0	191.0	1026.6
m43+sCD4	88.0	196.6	181.8	917.8
2F5	20.8	82.2	26.3	62.9
2F5+sCD4	26.5	77.3	31.7	48.9
4E10	19.3	25.1	76.9	116.4
4E10+sCD4	21.2	23.7	84.5	97.0

Each mAb was tested at 10 µg/ml concentration. (+): cleavage-competent gp160s, (−): cleavage-incompetent gp160s.

We then measured IgG_1_ m43 binding to HIV-1-infected cells, including C8166 cells acutely infected with the HIV-1 isolate BaL or K8 (R5-tropic), and H9 cells chronically infected with HIV-1 isolate NL4-3 (X4-tropic), and the effect of sCD4 on m43 binding to H9/NL4-3 cells in comparison with gp41-specific mAbs m44, 7B2 and 2F5 (Fig. 3B–C). M43 bound to the viral spikes of all three isolates on target cells although m43 binding to acutely infected C8166 cells was weaker than the binding of other mAbs (Fig. 3B). Unlike m44 and 7B2, whose binding to H9/NL4-3 cells was enhanced by sCD4, the binding of m43 was inhibited by sCD4 (Fig. 3C). We further characterized m43 for binding to H9/NL4-3 cells by competition flow cytometry with other HIV-1 mAbs (Fig. 3D). Unlike other gp120 and gp41 Abs, m43 inhibited the binding of b12, VRC01 and CD4-IgG_2_. M43 has little effect on the binding of other Abs to infected cells. These results suggest that m43 epitope is exposed in functional Env trimers and overlaps with the CD4bs.

### Neutralization activity of m43 against primary HIV-1 isolates and SHIVs

To characterize m43 for neutralization activity, we tested IgG_1_ m43 with primary HIV-1 isolates in cell line-based pseudovirus assays and in neutralization assays based on infection of PBMCs and measurement of p24 (PBMC-p24), or p27 (PBMC-p27), or RT (PBMC-RT) produced by infected cells. We first tested IgG_1_ m43 with a panel of 30 primary isolates from clade A, B, C, D and cross-clade AE and AG in PBMC-p24 assay and in TZM-bl pseudovirus assay in comparison with the CD4bs mAbs m14, m18, b12, CD4i mAb scFv m9, and gp41-specific mAbs m48, 2F5 and 4E10. HIV-1 infected patient sera pool 21.2 was included as a control in the assay (Table 3). Consistent with the observation reported previously, 2F5 and 4E10 were much more potent in TZM-bl assay than in PBMC assay. In contrast, IgG_1_ m43 neutralized the tier 2 or tier 3 viruses from each clade (A, B, C, D, AE, AG) tested in PBMC-p24 assay, but only two tier 1 virus from clade B and one tier 2 and one tier 3 viruses from clade D in TZM-bl assay. CD4i scFv m9 also showed increased potency in PBMC-p24 assays than in TZM-bl assays, which is consistent with the observation we reported previously [44]. TZM-bl cells have much higher coreceptor (CCR5 and CXCR4) surface density than PBMCs [44]. To investigate if m43 neutralization activity depends on the coreceptor density on target cells, we tested IgG_1_ m43 against a panel of clade B isolates in a pseudovirus assay using JC-10 (low CCR5) and JC-53 (high CCR5) as indicator cells in comparison with potent neutralizer VRC01 (Table 4). M43 exhibited higher inhibitory activity against JRFL (R5), JRCSF (R5) and 89.6 (R5X4) in JC-10 cells than in JC-53 cells, but lower inhibitory activity against BaL (R5) in JC-10 cells than in JC-53 cells and no difference in inhibiting entry of HxB2 (X4) into both cell lines, suggesting that the coreceptor density alone may not determine the neutralization activity of m43 in cell line-based assays.

**Table 3:** Percentage neutralization of IgG_1_s m43, m48, m14, m18, b12, 2F5 and 4E10 at 30 µg/ml against 30 HIV-1 primary isolates from different clades in TZM-bl and PBMC-p24 assays.

Virus	Clade	Tropism	TZM-bl assay									PBMC assay								
			m9	m14	m18	m43	m48	b12	2F5	4E10	21.2	m9	m14	m18	m43	m48	b12	2F5	4E10	21.2
92UG_029	A	X4	99	56	30	0	0	95	97	91	99	34	0	0	0	0	95	31	9	42
93RW_024	A	Dual	18	0	0	0	0	0	93	75	85	85	0	0	0	0	nd	66	0	38
00KE_KER2008	A	Dual	85	0	0	0	0	0	85	84	89	0	18	9	0	0	0	42	31	43
99KE_KNH1135	A	R5	14	0	11	20	3	100	92	83	73	96	29	32	64	69	95	81	93	75
00KE_KSM 4030	A	R5	0	0	0	0	0	0	97	95	91	72	72	49	77	32	31	81	72	85
89BZ_167	B	X4	0	84	100	100	0	95	100	99	99	0	61	91	43	12	70	70	60	99
92FR_BXO8	B	R5	98	84	83	86	0	85	92	89	98	99	37	46	1	19	70	51	23	99
90US_873	B	R5	93	30	18	22	0	100	99	93	96	79	0	45	60	47	93	96	89	99
96TH_NP1538	B	R5	69	0	0	0	0	0	61	44	89	94	0	4	18	19	nd	84	39	85
91US_4	B	R5	84	0	0	0	6	58	72	73	95	99	68	48	49	46	45	94	81	99
89SM_145	C	R5	86	24	9	6	15	0	0	90	83	52	4	8	45	0	nd	0	53	30
01TZ_911	C	R5	60	10	0	1	4	5	0	90	90	82	0	4	58	0	nd	0	0	29
98US_MSC5016	C	R5	83	70	40	31	0	0	18	98	87	95	0	0	0	0	nd	0	0	0
94IN_20635-4	C	R5	99	51	5	0	0	99	0	96	95	90	43	51	54	39	99	8	73	95
00TZ_A125	C	R5	16	0	0	0	10	0	0	83	54	42	42	27	50	24	64	39	36	53
00UG_D26830M4	D	R5	0	12	0	8	0	4	59	62	98	96	0	33	3	35		66	0	82
93UG_065	D	X4	99	21	18	16	6	5	99	90	78	96	67	45	73	28	19	70	62	64
99UG_AO8483M1	D	R5	90	87	86	88	10	100	74	59	97	99	36	52	25	0	99	86	62	83
98UG_57128	D	R5	94	65	60	65	16	100	1	70	86	92	60	40	59	26	98	10	56	50
00KE_NKU3006	D	R5	71	17	0	8	4	67	93	87	49	95	25	55	0	0	nd	74	34	37
90TH_CM235	AE	R5	19	24	0	23	24	22	91	80	70	26	0	11	0	0	nd	64	8	53
90TH_CM240	AE	R5	95	21	29	30	33	20	90	90	87	20	0	0	18	0	nd	10	37	9
96TH_NI1149	AE	R5	14	10	23	14	16	12	3	91	66	87	28	71	72	52	nd	19	47	92
96TH_M02138	AE	X4	0	21	33	25	0	16	99	97	90	2	0	0	0	0	nd	23	0	51
90TH_CM244	AE	R5	0	24	27	17	37	9	95	89	85	nd	nd	nd	nd	nd	nd	nd	nd	nd
02CM_1970LE	AG	R5	10	15	18	12	8	16	99	98	85	93	18	35	14	19	nd	72	0	40
98US_MSC5007	AG	R5	91	9	13	15	0	5	74	83	78	87	23	28	52	32	40	93	74	81
02CM_ 0013BBY	AG	R5	58	9	16	24	5	6	0	90	73	94	51	42	45	66	67	70	66	72
02CM_0015BBY	AG	R5	25	45	20	9	7	33	95	94	79	68	0	0	12	0	nd	48	11	45
02CM_0014BBY	AG	R5	36	12	6	5	13	19	19	91	83	93	8	53	1	19	nd	13	26	71
Percentage of virus neutralized			57	23	13	13	0	33	70	97	97	76	21	21	34	10	63	55	41	66

HIV-1-infected patient serum (pool 21.2) was included as a control. Percentage neutralization of viral infection are color coded so that the darker the color, the more potent the neutralization: a white box indicated <50% neutralization, a light grey box indicates 50%< percentage neutralization <90%, and a dark grey box indicates percentage neutralization >90%. n.d., not done.

**Table 4:** Percentage neutralization of IgG_1s_ m43 and VRC01 against a panel of clade B isolates by pseudovirus assay using JC-10 and JC-53 as indicator cells.

IC_50_ (µg/ml)	Clade	Tropism	JC-10 cells		JC-53 cells	
			m43	VRC01	m43	VRC01
Bal	B	R5	160.83	0.04	17.52	0.03
JRFL	B	R5	174.67	0.13	>200	0.14
JRCSF	B	R5	190.47	0.74	>200	1.18
89.6	B	Dual	5.67	1.2	28.28	1.24
HxB2	B	X4	0.2	0.07	0.2	0.1
VSV-G			>100	>10	>100	>10

**Figure 4:**
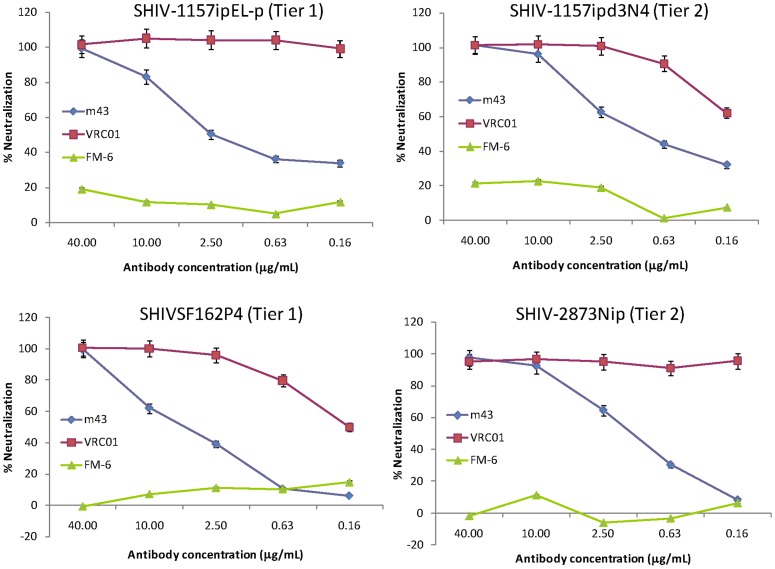
Percentage neutralization of IgG_1_ m43 with SHIV viruses. Three clade C (SHIV-1157ipd3N4, SHIV-1157ipEL-p, and SHIV-2873Nip) and one clade B (SHIV-SF162) SHIV viruses were tested in a PBMC-p27 assay. IgG_1_ VRC01 was used as positive control, while Fm-6 served as negative isotype control. The data shown are representative results obtained from two independent experiments.

We then tested IgG_1_ m43 with another panel of primary isolates from different clades by PBMC-RT assay in comparison with other three gp41-specific IgG_1_s m44, m48 and 4E10 (Table 5). IgG_1_ m43 neutralized all HIV-1 isolates tested in PBMC-RT assay except clade O isolate (BCF03). M43 exhibited neutralization activity that is comparable to or higher than that of m44, m48 and 4E10 against this panel of isolates by PBMC-RT assay (Table 5). We further tested IgG_1_ m43 with three clade C SHIV strains, the tier 2 SHIV-1157ipd3N4 [38] and SHIV-2873Nip [40] and the tier 1 SHIV-1157ipEL-p [39], and one clade B SHIV strain, the tier 1 SHIV_SF162P4_ [41], in a PBMC-p27 assay [38]. Because VRC01 is extremely potent (IC_50_ less than 0.16 µg/ml) against the four SHIVs, it was used as positive control, while Fm-6 served as negative isotype control. IgG_1_ m43 potently neutralized all SHIVs tested with IC_50_ values ranging from 0.7 to 4.9 µg/ml (Fig. 4). These results indicate that m43 targets a conserved epitope among the four SHIVs.

**Table 5:** Percentage neutralization of IgG_1_s m43, 44, 48 and 4E10 at 50 µg/ml against HIV-1 primary isolates from different clades in a PBMC-RT assay.

HIV-1 isolate	Clade	Coreceptor specificity	% Inhibition of RT activity (Mean ± SD)			
			m43	m44*	m48	4E10*
92UG029	A	X4	62+14	0+5.7	20+14	0+14
92HT594	B	X4R5	97+0.1	21+17	65+21	31+8.6
SHIV 89.6p	B	X4R5	96+0.4	96+0.3	83+12	25+13
92BR025	C	R5	66+3.7	72+1.6	68+1.6	58+3.2
97ZA003	C	R5	91+2.2	93+1.9	94+1.3	49+12
93IN101	C	R5	99+0.0	99+0.1	99+0.1	99+0.1
93MW959	C	R5	94+1.0	97+0.5	97+0.6	53+10
92UG001	D	X4R5	55+13	35+5.9	38+6.4	29+0.7
93TH073	E	R5	95+0.6	97+0.3	96+0.1	68+2.6
93BR029	F	R5	94+0.4	93+1.8	76+16	62+12
G3	G	R5	94+0.6	93+2.5	93+0.6	57+16
BCF03	O	R5	32+9.9	52+20	44+16	34+7.5
Median			94	93	87	51

The percentage inhibition of RT activity in the culture supernatant of HIV-1-infected PBMCs on day 7 is presented as a measure of the antibody inhibitory activity.

* : published data [33].

### Markedly different effects of IgG_1_ m43 and CD4 on functional Env trimer epitope exposure and on gp120 shedding

**Figure 5:**
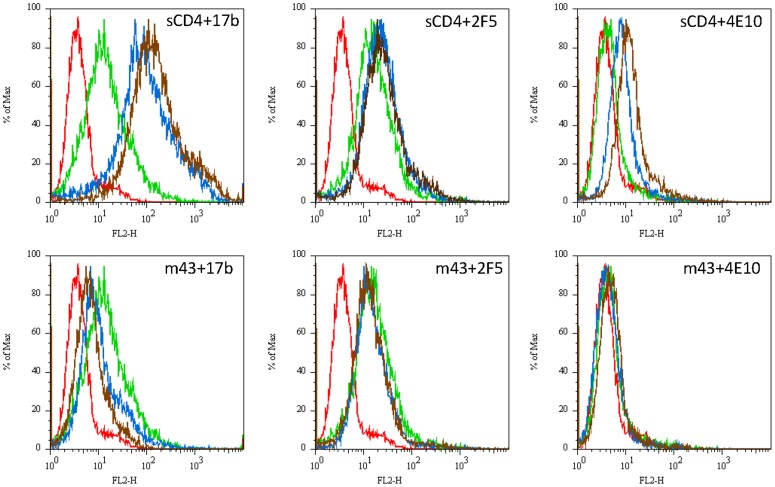
Effect of sCD4 (top panel) and m43 (low panel) on binding of 17b, 2F5 and 4E10 to JRFL gp160 on 293T cells by flow cytometry. Curves in red: 2nd Ab (PE-avidin) only; in green: 10 µg/ml biotinylated 17b, or 2F5, or 4E10; in blue: 10 µg/ml sCD4 or m43 in combination with 10 µg/ml biotinylated 17b, or 2F5, or 4E10; in brown: 50 µg/ml sCD4 or m43 in combination with 10 µg/ml biotinylated 17b, or 2F5, or 4E10.

**Figure 6:**
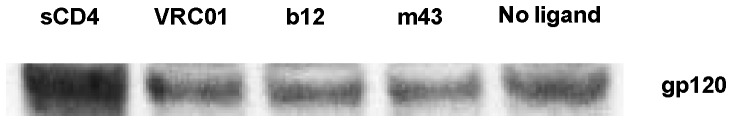
Effect of m43 binding on JRFL functional Env trimer. Western blot analysis of Env from the supernatant of 293T cells transfected with cleavage-competent gp160 JRFLdCT Env plasmid DNA. HIV-1 gp120 shedding upon binding of ligands, including sCD4, VRC01, b12, and m43, from the Env trimer on the surface of 293T cells was evaluated by probing the blot with anti-gp120 human mAb 39F (V3-specific). Each ligand concentration was 50 µg/ml.

To elucidate the mechanism of neutralization by m43, we investigated the effect of m43 on exposure of the coreceptor binding site and the MPER in the context of a functional Env trimer (Fig. 5). Unlike CD4, binding of m43 to cleavage-competent JRFL gp160 on 293T cells did not enhance the binding of CD4i mAb 17b and MPER-specific mAbs 2F5 and 4E10 to gp160. Instead, m43 inhibited 17b binding to the functional Env trimer. IgG1 m43 also slightly inhibited the binding of 2F5 to the functional Env trimer. 4E10 weakly bound to cleavage-competent JRFL gp160 and the effect of m43 on 4E10 binding was not obvious (Fig. 5). We further tested gp120 shedding upon m43 binding to cleavage-competent JRFL gp160 on 293T cells. Unlike CD4, m43 does not induce gp120 shedding upon binding, which is similar to the binding of VRC01 and b12 (Fig. 6). These observations suggest that binding of m43 to functional Env trimer may lock the Env in a non-fusogenic conformation, leading to inhibition of virus entry.

### Different binding patterns of IgG_1_s m43, VRC01 and b12 to cleavage-competent and cleavage-incompetent gp160s

**Figure 7:**
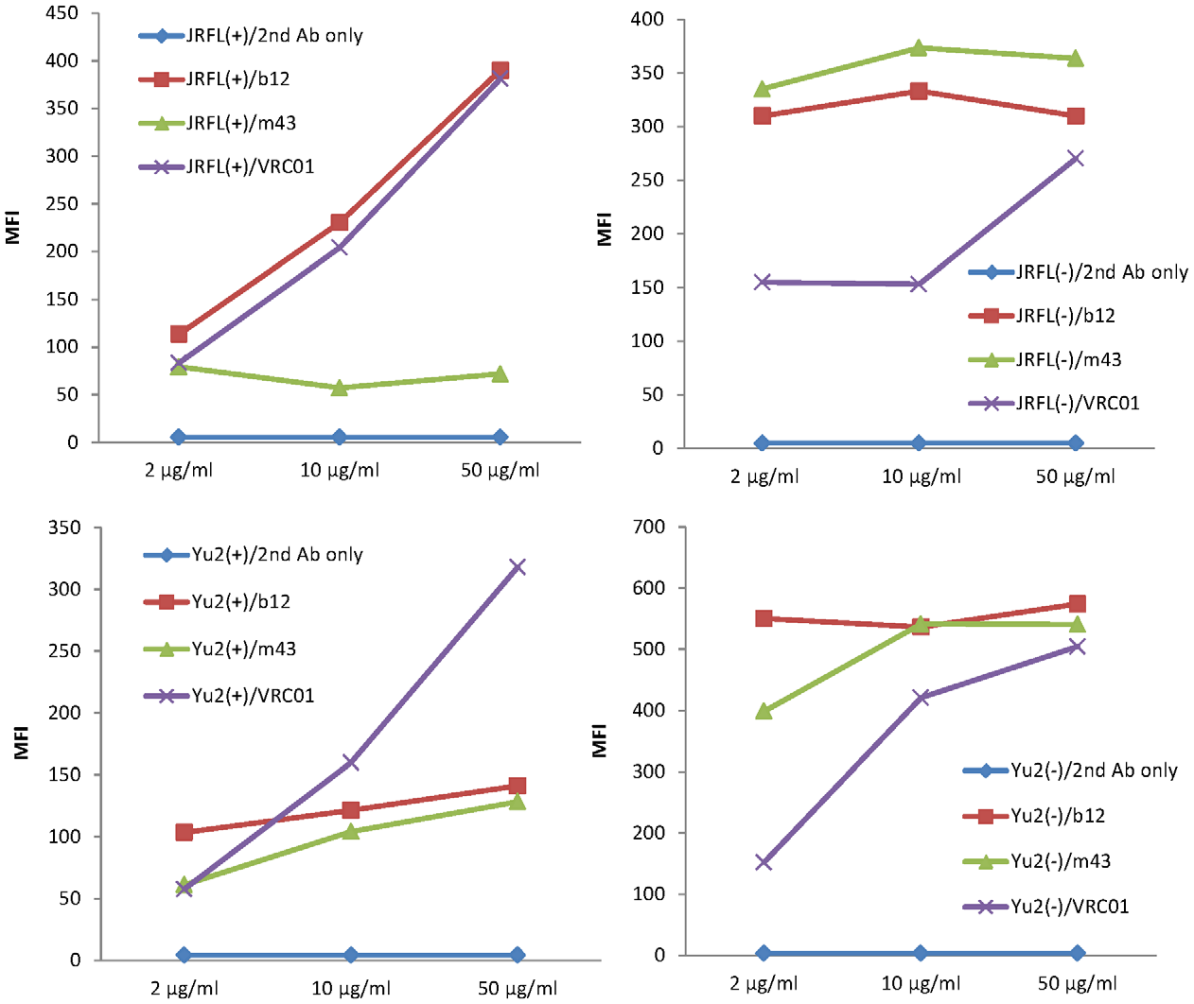
Binding of m43, b12 and VRC01 to cleavage-competent (+) and cleavage-incompetent (−) JRFL and Yu2 gp160s on 293T cells by flow cytometry. Different concentrations of mAbs used in the assay were indicated. PE conjugated to anti-human IgG, F(ab)2 were used as secondary antibody for detection.

When we measured the binding of IgG_1_ m43 to cleavage-competent JRFL gp160 in comparison with the binding of b12 and VRC01 by flow cytometry, we noticed that increased concentration of b12 and VRC01 led to dramatic increase in binding, while the binding of m43 was independent of m43 concentration. We then tested various concentrations of IgG_1_s m43, VRC01 and b12 for binding to cleavage-competent and cleavage-incompetent JRFL and Yu2 gp160s by flow cytometry (Fig. 7). It turned out that increased concentrations of VRC01 led to dramatically increased binding (2-4-fold increases in mean fluorescence intensity, MFI) of VRC01 to both cleavage-competent and cleavage-incompetent JRFL and Yu2 gp160s (Fig. 7). Increased concentrations of m43 and b12 also led to increased binding of m43 and b12 to cleavage-competent Yu2 gp160 (about 20% increases in MFI), but the increases with m43 and b12 were not as significant as that with VRC01. The binding pattern of b12 to cleavage-competent Yu2 gp160 was similar to that of m43, although b12 showed higher binding than m43 (Fig. 7). Both m43 and b12 showed high binding to cleavage-competent Yu2 gp160 and cleavage-incompetent JRFL and Yu2 gp160s, but their binding patterns were different. The binding of b12 to cleavage-competent Yu2 gp160 increased as the concentration of b12 increased, while the binding of b12 to cleavage-incompetent JRFL and Yu2 gp160s appeared to be independent of b12 concentration. The binding of m43 to cleavage-competent Yu2 gp160 increased as the concentration of m43 increased, while the binding of m43 to cleavage-incompetent JRFL and Yu2 gp160s increased as the concentration of m43 increased from 2 µg/ml to 10 µg/ml, but did not change as the concentration of m43 increased further (from 10 µg/ml to 50 µg/ml) (Fig. 7). The mechanisms for the different binding patterns of different mAbs remain to be elucidated.

## Discussion

Since the discovery of HIV-1 nearly three decades ago, an effective HIV-1 vaccine that can elicit bnAbs has yet to be developed. Identification of novel neutralizing determinants may help achieve this goal. We identified and extensively characterized a novel cross-reactive human mAb m43 that recognizes a new conserved neutralizing epitope shared by gp120 and gp41. Since the crystal structure of Env trimer is not available, we are not sure if m43 epitope is jointly formed by the CD4bs on gp120 and the N-trimer structure on gp41, or if m43 has dual specificity for one epitope on gp120 and another on gp41, which is very rare. Nevertheless, the m43 epitope is present on functional Env trimer and binding of m43 to viral Env prevents virus entry into target cells. Although m43 exhibited modest neutralization activity compared to potent bnmAb VRC01, it neutralized tier 1, 2 and 3 viruses from different clades and four SHIVs tested. M43 showed higher binding to cleavage-incompetent JRFL and Yu2 gp160s than to cleavage-competent JRFL and Yu2 gp160s, which may be attributed to the fact that uncleaved recombinant gp140s were used as antigens during the panning and screening for isolation of m43. Antibody engineering is in progress to improve m43 binding to cleavage-competent JRFL gp160. Improved dual binding to gp120 and gp41 may lead to broader and more potent neutralization activity of the antibody as exemplified by an engineered bispecific anti-HIV-1 antibody that can bind bivalently by virtue of one single chain antibody fragment (scFv) arm that binds to gp120 and a second arm to the gp41 subunit of gp160 [48]. Heterotypic bivalent binding enhanced neutralization compared with the parental antibodies. M43 may represent a new class of bnAbs and its epitope may be used for development of HIV-1 vaccine immunogens.

Characterization of the m43 epitope revealed that m43 bound to a conformational epitope that overlaps with the CD4bs on gp120 and the N-trimer structure on gp41. We tried to localize m43 epitope by panning a yeast-displayed Env fragment library against Fab m43, but no enrichment was observed (data not shown). CD4bs mAbs strongly competed with m43 for binding to recombinant gp140s and membrane-associated functional Env trimers (Fig. 2D and 3D), suggesting that m43 epitope overlaps with the CD4bs. The coreceptor binding site may not be involved in m43 binding as evidenced by lack of correlation between coreceptor density on target cells and m43 neutralization activity. The modest competition of CD4i mAbs with m43 for binding to the Env may be due to a steric hindrance. The effect of sCD4 on m43 binding to recombinant gp140s and membrane-associated Envs is different. sCD4 weakly inhibited m43 binding to recombinant gp140_89.6_ (Fig. 2C), while sCD4 enhanced m43 binding to cleavage-competent gp160_JRFL_ on 293T cells (Fig. 3A). However, CD4-IgG2 showed inhibitory effect on m43 binding to Env spikes on H9 cells infected with NL4-3 (X4-tropic) (Fig. 3C and 3D). This suggests that the m43 epitope on R5- and X4-tropic viral spikes may be different. We tested m43 binding to cleavage-competent gp160_HxB2_ (X4-tropic) on 293T cells by flow cytometry and observed that sCD4 also inhibited m43 binding to membrane-associated gp160_HxB2_ (data not shown). The mechanism for different effect of CD4 on m43 binding to R5- and X4-tropic viral Envs remains to be elucidated.

M43 may exist in infected individuals as indicated by a competition ELISA, in which m43 competed with polyclonal antibodies purified from immune sera of long-term nonprogressors for binding to coated Env trimers (data not shown). But the immune polyclonal antibodies are rich in CD4bs Abs, so the result was inconclusive. M43 was selected from a combinatorial antibody library (10^9^ individual clones) by phage display technology, in which the heavy chain and light chain are randomly paired. Nevertheless, since m43 has a high affinity (EC_50_ was below 10 nM for most of recombinant gp140s tested), it is reasonable to assume that m43 may have the original cognate heavy chain and light chain pairing [49], [50]. This is the first report of a new class of cross-reactive HIV-1-neutralizing mAbs cotargeting gp120 and gp41. We believe that the results from this study may have implications for vaccine development.

Strong competition of m43 with CD4bs mAbs for binding to recombinant Envs and functional Env trimer suggest that m43 neutralizes the virus by blocking the receptor and/or coreceptor binding to the Env. Unlike sCD4, binding of m43 to functional Env trimer does not enhance the binding of CD4i mAb 17b and MPER-specific mAbs 2F5 and 4E10, and does not induce gp120 shedding (Fig. 5 and 6), suggesting that m43 may neutralize the virus by a second mechanism which is to prevent Env conformational changes upon binding that is required for virus entry. There may be a third mechanism for m43 to neutralize the virus. Since m43 binds to the N-trimer structure, m43 may neutralize the virus by blocking a prefusion intermediate. We observed that b12 did not induce shedding in our current assay using cytoplamic tail truncated gp160_JRFL_, which is different from the result previously reported using full-length g160_JRFL_ in the assay [45].

## Conclusions

M43 recognizes a novel neutralizing determinant on HIV-1 Env that may involve the CD4bs of gp120 and the N-trimer structure of gp41. M43 epitope is conformational and conserved among HIV-1 primary isolates from different clades, and exposed on native Env spikes. M43 may represent a new class of bnAbs that target both gp120 and gp41 and neutralizes the virus by locking the native Env conformation, or by blocking the receptor and coreceptor binding to the Env, or by blocking a prefusion intermediate, or by two or all of three mechanisms. The novel neutralizing determinant of m43 and its possible mechanisms of neutralization may be useful for HIV-1 vaccine immunogen design.
